# Whole blood transcriptome analysis in amyotrophic lateral sclerosis: A biomarker study

**DOI:** 10.1371/journal.pone.0198874

**Published:** 2018-06-25

**Authors:** Wouter van Rheenen, Frank P. Diekstra, Oliver Harschnitz, Henk-Jan Westeneng, Kristel R. van Eijk, Christiaan G. J. Saris, Ewout J. N. Groen, Michael A. van Es, Hylke M. Blauw, Paul W. J. van Vught, Jan H. Veldink, Leonard H. van den Berg

**Affiliations:** 1 Department of Neurology, Brain Center Rudolf Magnus, University Medical Center Utrecht, Utrecht, the Netherlands; 2 Department of Translational Neuroscience, Brain Center Rudolf Magnus, University Medical Center Utrecht, Utrecht, the Netherlands; "INSERM", FRANCE

## Abstract

The biological pathways involved in amyotrophic lateral sclerosis (ALS) remain elusive and diagnostic decision-making can be challenging. Gene expression studies are valuable in overcoming such challenges since they can shed light on differentially regulated pathways and may ultimately identify valuable biomarkers. This two-stage transcriptome-wide study, including 397 ALS patients and 645 control subjects, identified 2,943 differentially expressed transcripts predominantly involved in RNA binding and intracellular transport. When batch effects between the two stages were overcome, three different models (support vector machines, nearest shrunken centroids, and LASSO) discriminated ALS patients from control subjects in the validation stage with high accuracy. The models’ accuracy reduced considerably when discriminating ALS from diseases that mimic ALS clinically (N = 75), nor could it predict survival. We here show that whole blood transcriptome profiles are able to reveal biological processes involved in ALS. Also, this study shows that using these profiles to differentiate between ALS and mimic syndromes will be challenging, even when taking batch effects in transcriptome data into account.

## Introduction

Amyotrophic lateral sclerosis (ALS) is a fatal neurodegenerative disease affecting motor neurons in the brain and spinal cord. Except for riluzole, there is no disease-modifying treatment and patients suffer from progressive paralysis that results in respiratory insufficiency within three to five years [1,2]. Twin studies estimate the heritability of ALS to be 0.61 (95% confidence interval = 0.38–0.78), indicating that genetic risk factors play an important role in ALS pathogenesis [3].

During the past years, a rapidly increasing number of genetic risk factors has been identified [4]. Based on these observations, multiple biological pathways have been linked to ALS including RNA processing, oxidative stress, mitochondrial dysfunction, excitoxicity, axonal transport and neuroinflammation [5,6]. Nevertheless, our understanding of ALS pathogenesis remains incomplete.

Furthermore, important questions in clinical practice still have to be answered. Diagnostic tests to confirm ALS are lacking: exclusion of alternative diagnoses remains essential but causes considerable diagnostic delay [2,7]. Previous studies, including sophisticated neuroimaging techniques, proteomic analyses in plasma and CSF, or gene expression profiling, have looked for diagnostic biomarkers, but challenges remain [8–13]. Observed patterns in biomarker research clearly require their predictive value to be assessed in well-defined patient cohorts, preferably including disease mimics, which are encountered in referral clinics.

Gene expression studies may satisfy both needs since they can shed light on differentially regulated pathways and identify valuable biomarkers [14,15]. Furthermore, whole blood gene expression profiles are easily obtained and thereby facilitate studies including a large number of cases and controls to improve the chance of finding a robust biomarker.

For these reasons, we used whole blood gene expression profiles in two large independent cohorts of ALS patients, healthy controls and ALS-mimics and show that these profiles reflect ALS pathophysiology. Using four different models we could reliably discriminate between ALS patients and controls. The road to a true diagnostic biomarker, however, proved challenging considering the marked batch effects and reduced accuracy when discriminating between ALS patients and ALS-mimics.

## Methods and material

### Patient selection

Patients were recruited from the out-patient clinic specialized in motor neuron diseases at the University Medical Center Utrecht, The Netherlands. ALS patients fulfilled the revised El Escorial criteria for possible, probable (lab supported) or definite ALS [16]. Both patient with and without a family history for ALS or frontotemporal dementia participated in this study. Age at onset was defined as age at appearance of first muscle weakness, difficulty speaking or swallowing. Survival was defined as time from disease onset to death, tracheostomy or non-invasive ventilation >23 hours a day. Survival status of patients is regularly checked through the Dutch Municipal Personal Record Database and ALS care teams. The date of the last check served as the censoring date in survival analyses. The controls were population-based subjects, matched for gender and age, free of any neuromuscular disease [17]. Finally, the 75 ALS-mimics were patients who were referred to our out-patient clinic for motor neuron disease in whom ALS was suspected. After reviewing the patient’s history, neurological examination, additional diagnostic tests and considering the course of the disease, one or more neurologists specialized in motor neuron diseases made an alternative diagnosis (Table A in S1 File). Patients with primary lateral sclerosis and progressive muscular atrophy were excluded. All participants gave written informed consent and the Medical Ethics Committee at the University Medical Center Utrecht approved this study.

### RNA isolation and quality control

In the patient group, blood was drawn in the morning on the day they were seen at our clinic because of suspected ALS or within 60 days after the initial visit. Control subjects’ blood was also obtained in the morning. Venous blood was collected in PAXgene tubes containing reagents that immediately stabilize messengerRNA (mRNA). After maintaining samples at room temperature for 2 hours, tubes were stored at -20 °C. For isolation and purification of mRNA, PAXgene extraction kits (Qiagen) were used according to the manufacturer’s protocol. This included purification by DNAse treatment. Globin reduction treatments were not performed. Quality of isolated RNA was assessed using the Agilent 2100 Bioanalyzer system. Samples with RNA Integrity Number (RIN) values < 7 were dismissed. Furthermore, quality was assessed by visual inspection of gel electrophoresis patterns.

### Gene expression profiling

Before RNA hybridization, samples were randomized to avoid batch effects correlated to the diagnosis. Samples were hybridized to two different platforms at two different laboratories: Illumina’s HumanHT-12 version 3 and version 4 BeadChips according to manufacturer’s protocol (Illumina, Inc., San Diego, CA, U.S.A.).

### Quality control—Data preparation

Final Reports from Illumina’s GenomeStudio were imported in R (http://cran.r-project.org). After quantile normalization, gender was checked by expression of gender-specific Y chromosomal probes (*JARID1D* and *RPS4Y1*). Samples with gender mismatches were excluded. Subsequently expression values were log_2_ transformed and quantile normalized. Arrays were projected along the first and second principal component and outliers were dismissed. This resulted in the exclusion of 34 patients and 33 control subjects from the training set and exclusion of 13 patients and 7 control subjects from the test-validation set.

High quality probes were selected, including true autosomal probes only. Using the BLAT function in UCSC’s Genome Browser, all probes were aligned to the NCBI reference genome build 36. Probes with multiple BLAT hits with a sequence homology of > 95% were defined as aspecific and excluded from further analysis. We looked for retired probes using the RefSeq and UniGene (build #228) databases. Retired probes were excluded from further analysis. Finally, probes with identical probe IDs that overlapped between the Illumina HumanHT12 v3 and v4 platform were selected.

### Surrogate variable analysis

To eliminate expression heterogeneity caused by known and unknown technical and biological background, data from both training and test sets were combined and normalized, applying surrogate variable analysis (SVA). This produces surrogate variables for which expression levels can be corrected by calculating residuals in a linear regression model. In previous studies, this correction has been proven to reduce batch-sp3ecific background noise, thereby increasing the ability to detect biologically meaningful signals [18].

### Differential expression

To find differentially expressed genes in a two-stage (discovery-replication) design, we used the larger cohort hybridized to the IlluminaHT-12 v3 BeadChip in the discovery phase and that hybridized to the IlluminaHT-12 v4 BeadChip in the replication phase. Because the combination of p-values with the fold change to determine differential expression has proven more robust than p-values alone, we combined both parameters to define differential expression [19]. The p-values were calculated applying linear regression corrected for age, gender, riluzole use and the surrogate variables. For replication purposes, genes that showed differential expression (p < 0.05 unadjusted for multiple testing) and a 1.5-fold median change were taken to the replication phase. In the replication phase transcripts were considered differentially expressed when the met 2 criteria:

- An FDR corrected p-value < 0.05 from linear regression.
- A 1.5-fold change in median expression values between ALS cases and controls.

For these genes, enrichment of gene ontology processes and KEGG pathways was examined using DAVID Bioinformatics Resources 6.7 (http://david.abcc.ncifcrf.gov) [20]. Furthermore, we performed tissue enrichment analysis for differentially expressed genes using FUMA [21]. For both functional and tissue enrichment analysis we used all probes passing quality control as background.

Next, we assessed whether subgroups of ALS patients exhibit distinct gene expression patterns. For this purpose, we split the group of ALS patients based on site of onset (bulbar vs. spinal onset) and *C9orf72* status (wild-type vs. expanded) and split the control cohort into two proportionally sized cohorts. We applied surrogate variable analysis with 3 phenotype groups: bulbar, spinal and control for site of onset and wild-type, expanded and control for *C9orf72* status. In the SVA-corrected data we compared the effect-estimates and p-values for differentially expression between both subgroups (bulbar vs. spinal onset and *C9orf72* wild-type vs. expanded).

### Predicting disease status

We used four different methods to train models that could be used to predict disease status: linear discriminant analysis (LDA), support vector machines (SVM) [22], nearest shrunken centroid (NSC) [15] and least absolute shrinkage and selection operator (LASSO) [23]. These approaches are available through the R packages MASS, e1071, pamr and glmnet respectively (cran.r-project.org).

**Fig 1:**
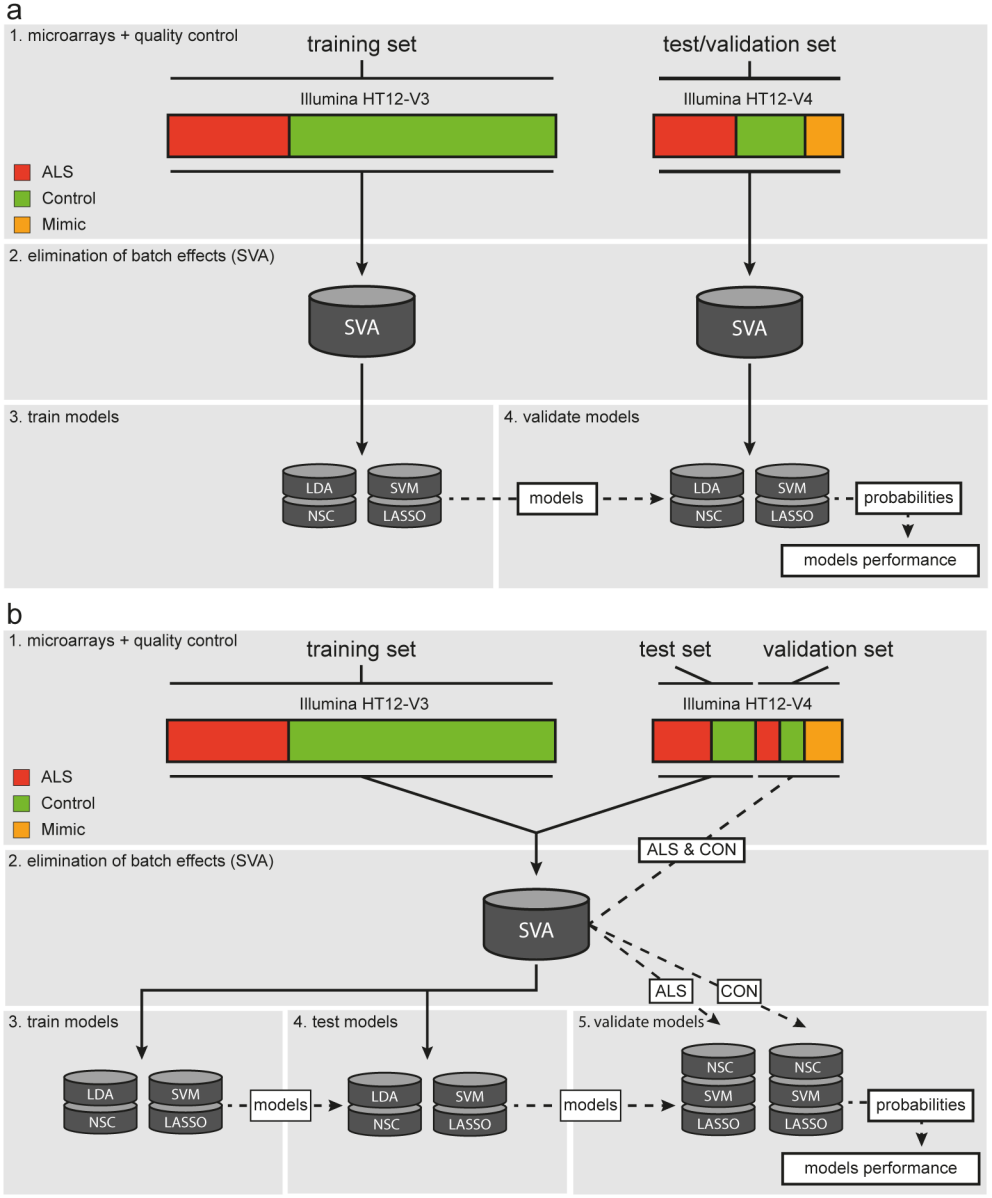
Procedures for training, testing and validation of the classifiers. **(a)** In the first approach the training and test/validation set were treated as totally separate sets. **(b)** In the second approach batch effects between the training and test set were overcome by surrogate variable analysis, after which the sets were separated and the models were trained and tested. The samples in the validation set were corrected using the surrogate variables twice, labelled as ALS and as control, before assessing the performance of the models.

In a biomarker approach, the trained model is ideally validated in an external, independent cohort. To keep the test/validation set totally independent, we eliminated batch effects by SVA on the training and test/validation set separately. Subsequently, the models were trained on the training set and their performance was assessed in the independent test/validation set (Fig 1a). This approach, however, can suffer from batch effects between the training set and test/validation set that are not captured by SVA since both sets were not combined. As a consequence, the chances of externally validating the model are limited. We therefore took a second approach, where we combined the training and test set to remove batch effects, by applying SVA on this dataset as a whole (Fig 1b). The training and test set were subsequently separated again before training and testing the model. Using this approach, however, the test set can no longer be considered an independent validation set. To approximate an independent dataset, we used the validation set, that included samples that were not used in the SVA but were hybridized in the same batch as the test set. These validation samples were normalized using the surrogate variables derived from the training and test set. For SVA, labelling samples as ALS or control subject *a priori* is essential for normalization. This entails the risk of overestimating the prediction model’s performance and in a clinical practice this label would be unknown. Therefore, the validation samples were normalized twice, as ALS sample and as control, regardless of their true class. The highest probability for each class, defined by the models, was chosen as the definite class prediction. Likewise, the mimics were also included in this validation set.

### Predicting survival

To predict survival in patients, a previously described modification of the nearest shrunken centroid algorithm was used [24]. This algorithm uses the probes that were associated with survival in a Cox proportional hazards model corrected for gender, age at onset and site of onset (spinal versus bulbar) in the training set. Based on expression profiles of these probes, patients in the training set were clustered into two groups: those with long survival and those with short survival. Subsequently, the NSC prediction model was developed using the survival-associated probes in these two groups after which this model classified samples in the test set as long or short survivor. We assessed the performance of this prediction model by testing the association between the predicted survival class (long vs. short) and true survival in a Cox proportional hazards model.

## Results

### Study population

Baseline characteristics for the 397 ALS patients, 645 control subjects and 75 ALS-mimics that passed quality control were virtually identical between the different sets (Table 1). Patients with diseases mimicking ALS were more frequently male and were younger than ALS patients and controls. The spectrum of diagnoses in the ALS-mimics reflected the clinical practice of our tertiary referral center for motor neuron diseases (Table A in S1 File).

**Table 1:** Baseline characteristics.

	Training set		Test set		Validation set		
	*ALS*	*Controls*	*ALS*	*Controls*	*ALS*	*Controls*	*Mimics*
N	233	508	114	87	50	50	75
Gender (% female)	38.6	44.7	41.2	44.8	42	38	23
*C9orf72* (%)	5.2	-	14.9*	-	8.0	-	-
Age (IQR)	64.7 (57–72)	62.9 (57–69)	61.1 (55–68)	62.4 (57–69)	64.5 (55–69)	60.0 (55–65)	57.9 (47–64)
Age at onset (IQR)	63.9 (56–71)		61.8 (55–71)		64.9 (55–70)		
Survival (IQR)	31.3 (21–36)		27.0 (18–40)		30.5 (24–40)		
Bulbar (%)	38.6		39.5		22.0		
Platform	HT-12 V3		HT-12 V4		HT-12 V4		

C9orf72 was not tested in controls or ALS mimics. Median follow-up for survival was 4.4 years (min 1.8 years, max 9.7 years).

* p = 0.004, χ^2^-test comparing training and test set.

IQR = interquartile range, HT-12 = Illumina HumanHT-12 expression array

### Data preparation

**Fig 2:**
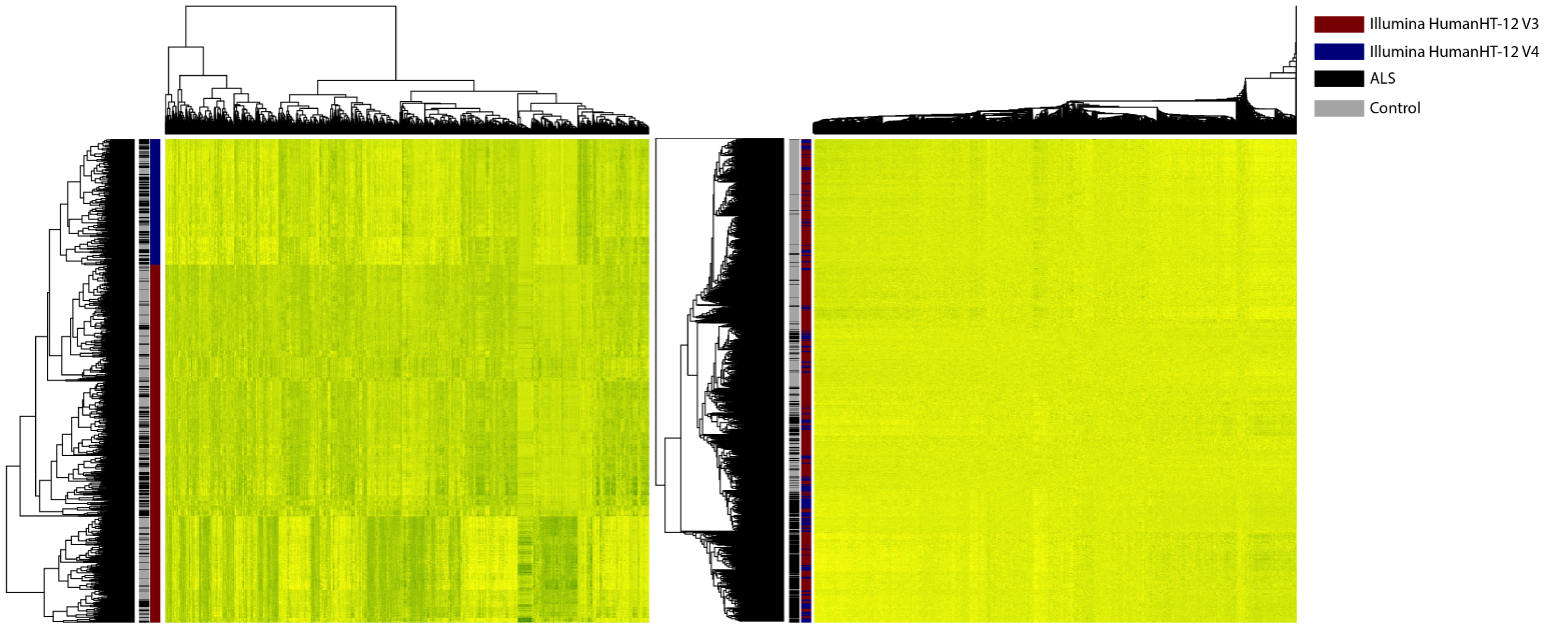
Elimination of expression heterogeneity by surrogate variable analysis. The left heatmap displays the expression of the 5,000 most variable probes before correction by surrogate variable analysis. The right heatmap displays the expression of the 5,000 probes after correction by surrogate variable analysis. Rows display arrays and columns reflect probes. Arrays are clustered by hierarchical clustering. Black lines reflect patients and grey lines control subject. Red lines display array hybridized on Illumina’s HumanHT-12 version 3 BeadChips and blue lines those hybridized on version 4. Before SVA correction, arrays are perfectly clustered based on the platform used: after SVA correction, these batch effects are corrected for.

In total, 29,830 unique, autosomal, non-retired probes were present on both the Illumina HumanHT-12 v3 and v4 BeadChip and were suitable for further analysis. Correction for expression heterogeneity, reflecting the technical and biological background (especially batch effects) by surrogate variable analysis (SVA) of our training and test set, resulted in 34 surrogate variables. Fig 2 displays the elimination of batch effects that dominated expression profiles before SVA correction.

### Differential expression analysis reveals pathways involved in ALS pathogenesis

Linear regression identified 7,038 genes that were expressed differentially between patients and control subjects in the discovery set. Of these genes, 2,943 were expressed differentially in the replication set (FDR corrected p-value < 0.05) with at least a 1.5-fold change between patients and control subjects. Gene Ontology (GO) analysis of molecular functions identified *RNA binding* (*P* = 8.61 × 10^−9^, FDR corrected *P* = 1.43 × 10^−5^) and *enzyme binding* (*P* = 1.20 × 10^−5^, FDR corrected p = 1.99 × 10^−2^) as the most enriched molecular functions; other molecular functions were not significantly enriched after FDR correction for multiple testing (Table 2). The biological process, *intracellular transport* (*P* = 4.01 × 10^−8^, FDR corrected *P* = 7.47 × 10^−5^), was most significantly enriched after correction for multiple testing. Furthermore, the GO biological process *programmed cell death* was significantly enriched for genes differentially expressed between ALS patients and healthy controls (*P* = 7.53 × 10^−7^, FDR corrected *P* = 1.40 × 10^−3^). Other enriched processes were closely related to either intracellular transport or programmed cell death (Table 2). Finally, KEGG (Kyoto Encyclopedia of Genes and Genomes) enrichment analysis did not identify any significantly enriched pathways after FDR correction. We found the differentially expressed genes most highly expressed in blood and spleen tissue compared to the other 30 general tissues studied in GTEx (Fig A in S1 File). This might reflect the increase in power to detect differentially expressed genes in our whole blood expression profiles for genes with overall higher expression levels in whole blood. A full list of differentially expressed genes is provided in S1 Table.

**Table 2:** Pathway analysis.

**Gene Ontology—Molecular Function**				
*Term*	*p-value*	*Fold Enrichment*	*FDR*	*N*
RNA binding	8.61 × 10^−9^	1.57	1.43 × 10^−5^	150
Enzyme binding	1.20 × 10^−5^	1.51	0.02	105
**Gene Ontology—Biological Processes**				
*Term*	*p-value*	*Fold Enrichment*	*FDR*	*N*
Intracellular transport	4.01 × 10^−8^	1.56	7.47 × 10^−5^	140
Protein transport	5.60 × 10^−8^	1.51	1.04 × 10^−4^	157
Establishment of protein localization	6.12 × 10^−8^	1.50	1.14 × 10^−4^	158
Programmed cell death	7.53 × 10^−7^	1.52	1.40 × 10^−3^	127
Protein localization	2.28 × 10^−6^	1.40	4.25 × 10^−3^	169
Apoptosis	4.76 × 10^−6^	1.48	8.88 × 10^−3^	122
Vesicle-mediated transport	6.88 × 10^−6^	1.49	0.01	117
Cell death	2.00 × 10^−5^	1.41	0.04	138

Top enriched Gene Ontology terms for differentially expressed genes. FDR: false discovery rate, N: number of differentially expressed genes per category.

We next studied the sub-phenotypes of ALS by dividing our patient cohort based on site of onset (spinal vs. bulbar) of *C9orf72* status (wild-type vs. expanded). Here, we found no evidence for heterogeneity in gene expression profiles between the sub-phenotypes as the changes in gene expression compared to (independent) controls were highly correlated (Fig B in S1 File).

**Fig 3:**
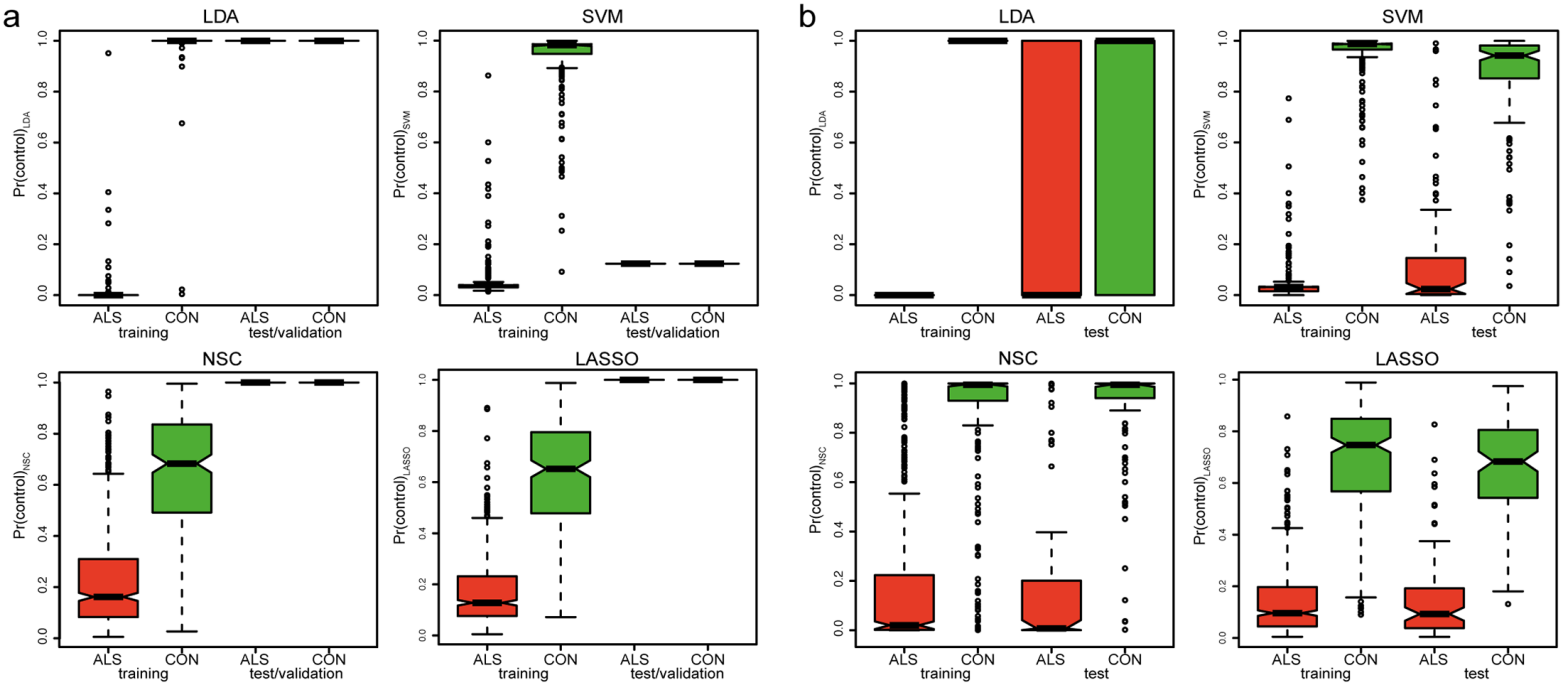
Probabilities for training and test/validation set. Boxplots of probabilities given by the four different models (LDA, SVM, NSC and LASSO) in the training and test/validation set for approach 1 (**a**) and approach 2 (**b**).

Through the first approach (Fig 1a) we trained the models (LDA, SVM, NSC and LASSO) on the training set in which they were able to discriminate between cases and controls. Nevertheless, none of the models could discriminate between cases and controls in the test/validation set, due to severe batch effects between the training and test/validation set (Fig 3a). Therefore, the first approach that treated the test/validation set as a totally independent dataset did not yield a reliable biomarker.

**Fig 4:**
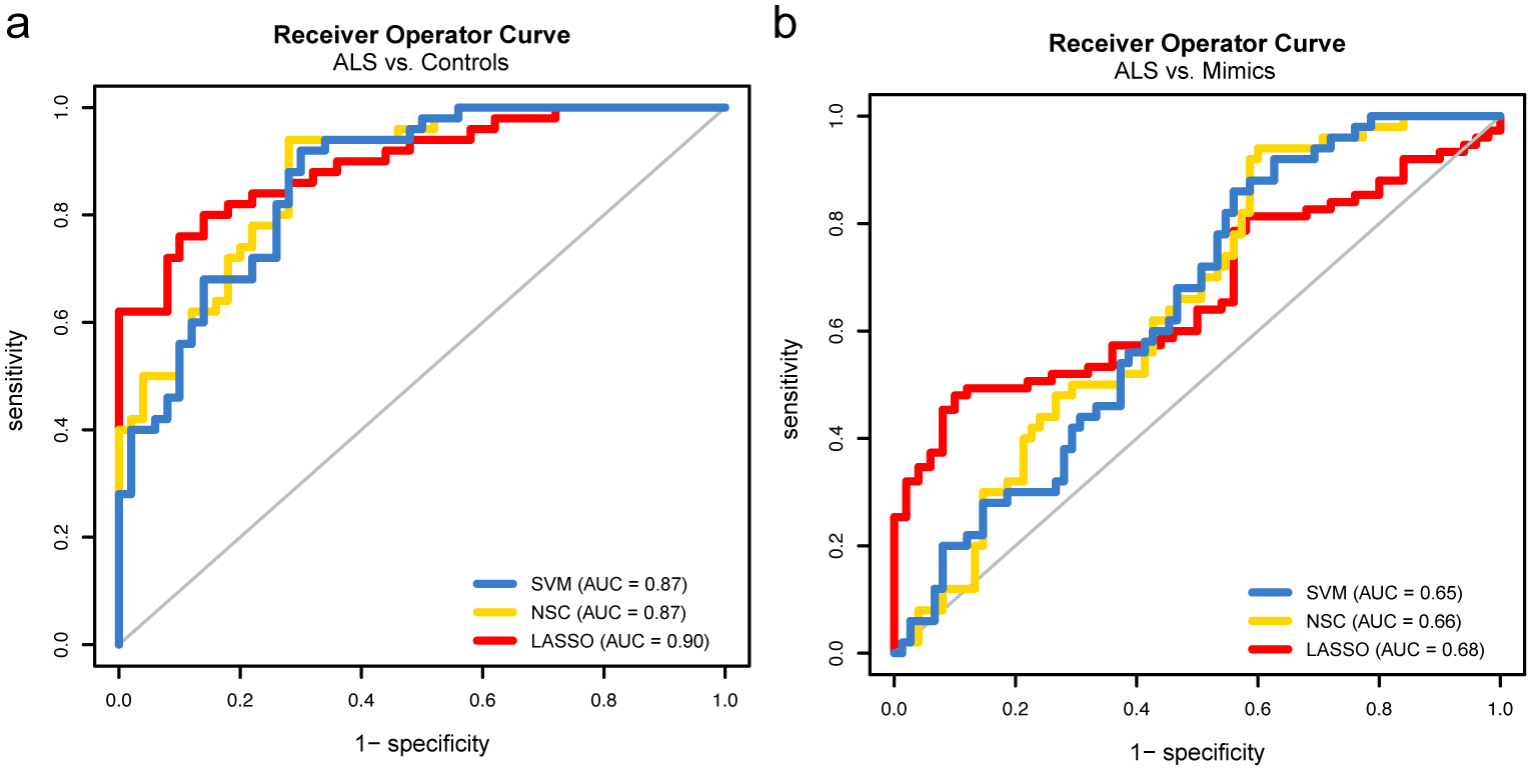
Receiver operator curves for validation set. (**a**) Receiver operator curves for the SVM, NSC and LASSO classifiers in the validation set when discriminating between ALS cases from controls and (**b**) discriminating ALS cases from ALS-mimics.

To overcome the batch effects between the training and test/validation set we took approach 2 that applied SVA on the training and test set combined, prior to training the models (Fig 1b). Except for LDA, this approach improved discrimination between ALS cases and controls in the test set (Fig 3b). Shrinking of effect estimates left 106 and 77 informative genes for NSC and LASSO respectively. All models, except LDA, were taken to the validation phase where they were tested to predict the class of 50 ALS cases and 50 controls. Whereas all classifiers performed well in the validation phase, the resulting receiver operator curves indicated the LASSO model performed best (area under curve = 0.90, Fig 4a). We next assessed whether the label used for SVA (ALS or control) in the validation set could have resulted in biased predictions. Overall the probabilities were highly correlated with some evidence for bias in the SVM and NSC classifiers (*R*^2^ = 0.89 for both), but not for LASSO (*R*^*2*^ = 0.97, Fig C in S1 File). In contrast to the high accuracy obtained when discriminating ALS patients from healthy controls, none of the models performed well when they were tested in the set of 50 ALS cases and 75 ALS-mimics (area under curve 0.65–0.68, Fig 4b).

### Gene expression profiles do not serve as a biomarker for survival

**Fig 5:**
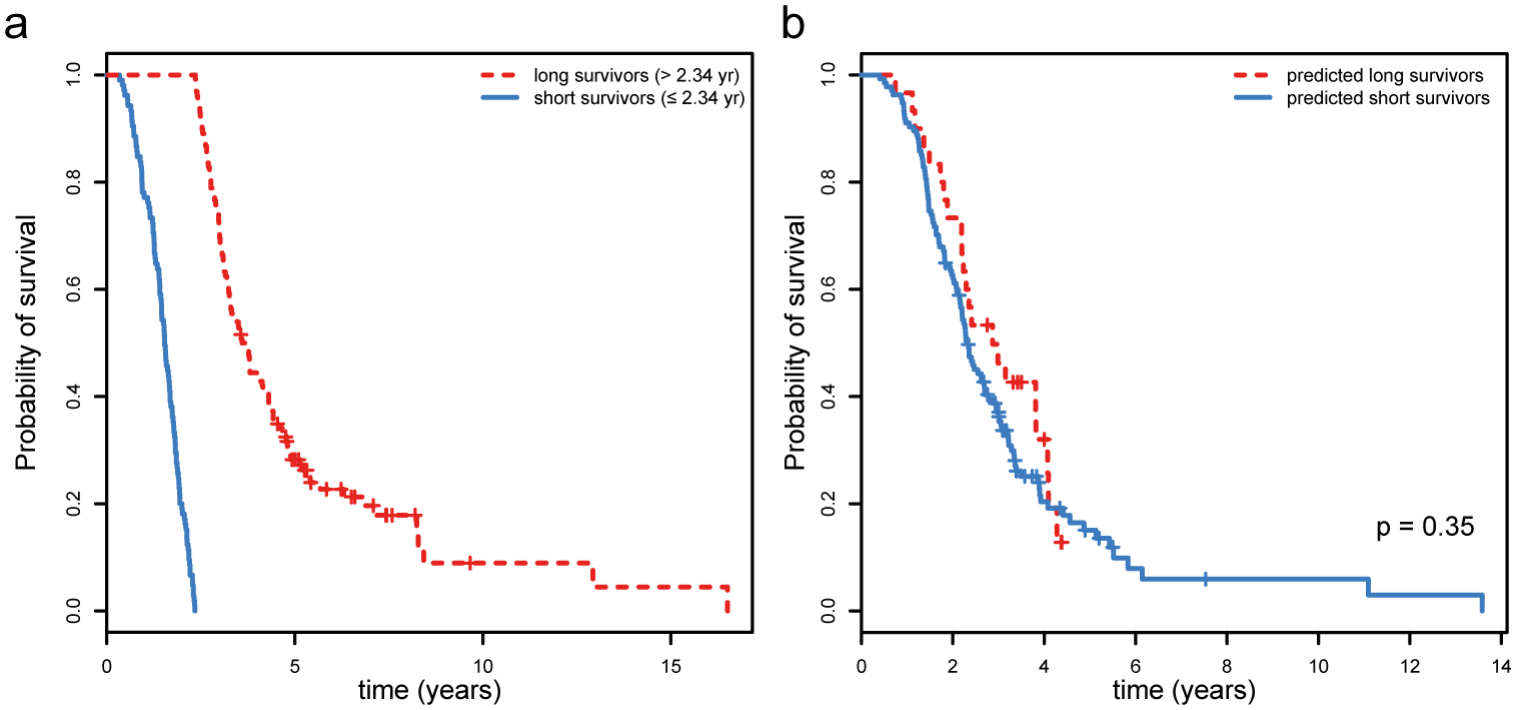
Survival curves for predicted survival classes. (**a**) Differences in survival time for the so-termed “long survivors” and “short survivors” in the training set, which was used as input to train the nearest shrunken centroid survival model. (**b**) The differences in true survival between the predicted “long survivors” and predicted “short survivors” in the test set.

In the training set, expression of 2,324 genes was significantly associated with survival in a Cox proportional hazards model corrected for gender, age at onset and site of onset. We then trained a survival prediction model applying the NSC algorithm including the probes associated with survival in the training set. The prediction model divided patients in the training set into two groups: those with long and those with short survival (cut-off = 2.34 years, Fig 5a). In the test set, the actual survival time for the predicted long survivors was, however, virtually similar to the survival time in the predicted short survivors (median = 2.82 and 2.28 years respectively, p = 0.35, Fig 5b). This means the expression of any subset of these 2,324 genes was not sufficiently informative to predict survival for ALS patients.

## Discussion

Using transcriptome-wide analyses, assessing differential expression, we have shown that whole blood mRNA profiles were able to detect pathways involved in motor neuron pathophysiology in ALS patients. We found 2,943 genes to be differentially expressed, which were predominantly involved in RNA processing and cellular transport, which are key pathways in ALS pathogenesis [6]. In an effort to find a clinically useful diagnostic biomarker, we applied four different algorithms in two different approaches including ALS patients, healthy controls and ALS-mimics. After correction for severe batch effects, the models could discriminate well between ALS patients and healthy controls. We subsequently assessed its performance including ALS-mimics to represent a tertiary referral clinical setting, a crucial step that is often omitted in biomarker research. Unfortunately, the models failed to distinguish between ALS patients and ALS-mimics, nor could we predict survival.

RNA processing is one of the best-established pathways involved in ALS pathogenesis. Common causes of familial ALS, including *TARDBP* and *FUS* mutations, as well as polyglutamine repeat expansions in *ATXN2* and *SMN1* duplications as susceptibility factors for sporadic ALS, all play an important role in multiple aspects of RNA processing [25–28]. Furthermore, RNA foci are observed in cells harboring the *C9orf72* repeat expansion. It has been hypothesized that this leads to RNA-mediated toxicity caused by sequestering of RNA binding proteins in these foci [29,30]. Alternatively, RAN translation of this repeat can lead to nucleolar stress and neurodegeneration via the suppression of ribosomal RNA synthesis [31]. We did not find any of the known ALS-associated genes involved in RNA binding to be differentially expressed. Nevertheless, we did find *TARBP2* and *HNRNPA(0/B)*, genes closely related to *TARDBP* and *FUS* respectively, to be differentially expressed. Other differentially expressed gene families involved in RNA processing were ribosomal proteins (*RPL4*, *RPL5*, *RPL8*, *RPL15*, *RPL19*, *RPL22*, *RPLP0*, *RPLP1*, *RPLP2*, *RPS13*, *RPS15A*, *RPS25*, *RPUSD4*), DEAD-box proteins (*DDX17*, *DDX19A*, *DDX19B*, *DDX21*, *DDX24*, *DDX31*, *DDX50*, *DDX51)* and eukariotic translation initiation factors (*EIF2A*, *EIF2C1*, *EIF3B*, *EIF4A2*, *EIF4A3*, *EIF5A)*. Interestingly, a recent study showed that, although gene expressions profiles can be highly tissue-spedific [32], many genes involved in RNA processing were differentially expressed in fibroblasts [33]. This, together with our observations, suggests that gene expression profiles in non-neuronal tissue can reflect motor neuron pathology in ALS patients. The identification of RNA processing using an unbiased approach underlines its importance in ALS pathogenesis, and the potential for whole blood gene expression profiles to highlight motor neuron pathology.

Apart from RNA processing, we found genes involved in intracellular transport to be differentially expressed. As motor neurons are highly polarized, intracellular transport—and specifically axonal transport—is crucial to maintain their function. In *SOD1*^*G93A*^ transgenic mice, impaired axonal transport precedes symptomatic muscle weakness suggesting this might be an early feature of ALS pathology [34]. Furthermore, *VAPB* mutations have been shown to impair axonal transport of mitochondria, a finding also observed in *SOD1*^*G93A*^ transgenic mice [35]. Finally, ALS-specific mutations in *TARDBP* impair anterograde axonal transport of mRNA in drosophila and mouse models [36]. These observations corroborate the premise that cellular transport plays a crucial role in ALS pathogenesis.

We have shown batch effects between hybridization platforms and laboratories severely challenge the development of a reliable biomarker that uses gene-expression microarrays. When both our datasets were combined, however, and batch effects were corrected for, three different models were able to differentiate accurately between patients and healthy controls. We note that the performance of our prediction models was not affected by the difference in *C9orf72* carriers between the training and testing set. This indicates that whole blood gene expression profiles harbour information that might ultimately be used as a diagnostic biomarker. Although this seems promising, important challenges need to be overcome.

First, the models should be robust to batch effects so they can be externally validated. We did not achieve this in our original approach and it is therefore possible that the models we have developed in our second approach will not perform well in an external dataset. The batch effects, however, are almost inherent to the technique used for microarray hybridization [37]. Therefore, alternative techniques such as RNAseq can be used. Whereas RNAseq also suffers from batch effects, many strategies have been developed to correct for these batch effects internally (normalization within a batch), which may ultimately yield better normalized signals than those obtained through microarrays [38]. Considering that gene expression profiles can tissue-specific^32^, another strategy is to obtain a better signal to noise ratio, is to study the primarily affect tissue. The ability to study primarily affected tissue has been responsible for the success of microarray-based gene expression biomarkers in the cancer field [14,39,40]. To obtain neuronal tissue from ALS patients and controls, however, will limit the sample size for biomarker discovery and raises ethical questions when applied in a clinical diagnostic setting.

The second challenge that needs to be overcome before a biomarker can be applied in the clinic, is that it should be able to discriminate between ALS patients and ALS-mimics. This crucial step is often omitted in biomarker studies. Whereas our models performed well when in a case-control setting, they did not discriminate well between ALS cases and mimics. One possible explanation is that some ALS-mimics not only resemble ALS clinically, but also exhibit a similar gene expression profile. This could be caused by shared pathways in disease etiology, as is seen among neurodegenerative diseases [41]. Alternatively, gene expression profiles of ALS patients may partly reflect secondary mechanisms caused by acquired muscle weakness, also present in ALS-mimics. Future studies should therefore include an even larger number of ALS-mimics, so even more subtle gene expression changes between ALS patients and ALS-mimics can be picked up when training the models.

Finally, changes in whole blood-derived gene expression profiles are easily obtained and, as we have shown, in part reflect motor neuron biology in ALS. Alternative ways to follow up these observations, include studying longitudinal gene expression measurements in ALS patients. These profiles, easily collected in clinical trials, can shed light on changes in perturbed biological processes during the disease course. Furthermore, developing an easily obtained biomarker, that can monitor disease progression is highly warranted for effective trial design.

In conclusion, we have shown that whole blood transcriptome profiles are able to reveal biological processes involved in ALS. Also, this study shows that using these profiles to differentiate between ALS and mimic syndromes will be challenging, even when taking batch effects in transcriptome data into account.

## Supporting information

S1 File **(Table A) Diagnoses for ALS mimics. (Fig A) Tissue enrichment for differentially expressed genes**. Top: p-values for differentially upregulated genes per tissue when compared to expression levels in all other tissues. Middle: p-values for differentially downregulated genes per tissue when compared to expression levels in all other tissues. Bottom: p-values for differentially expressed (up- and downregulated) genes per tissue when compared to expression levels in all other tissues. **(Fig B) Comparison of differentially expressed genes for sub-phenotypes**. Top: comparison of differential expression between spinal onset ALS vs. controls and bulbar onset ALS vs. controls. Bottom: comparison of differential expression between ALS-C9orf72-wild-type vs. controls and bulbar onset ALS-C9orf72-expanded vs. controls. **(Fig C) Correlation of posteriors after SVA correction**. Correlation of probabilities in the validation set for NSC, SVM and LASSO after SVA correction labelled as ALS (x-axis) or control (y-axis). Whereas the probabilities for all classifiers where highly correlated, LASSO was free of any bias introduced by the SVA label. The top row displays the results for ALS vs. controls and the bottom row for ALS vs. mimics.
(DOCX)

S1 Table List of differentially expressed genes. (XLSX)
