# Supplementary material for: Whole blood transcriptome analysis in amyotrophic lateral sclerosis: A biomarker study
Source: PLoS One. 2018 Jun 25;13(6):e0198874. doi: 10.1371/journal.pone.0198874 (PMC6016933; doi:10.1371/journal.pone.0198874)
Supplement: S1 File — (Table A) Diagnoses for ALS mimics. (Fig A) Tissue enrichment for differentially expressed genes. Top: p-values for differentially upregulated genes per tissue when compared to expression levels in all other tissues. Middle: p-values for differentially downregulated genes per tissue when compared to expression levels in all other tissues. Bottom: p-values for differentially expressed (up- and downregulated) genes per tissue when compared to expression levels in all other tissues. (Fig B) Comparison of differentially expressed genes for sub-phenotypes. Top: comparison of differential expression between spinal onset ALS vs. controls and bulbar onset ALS vs. controls. Bottom: comparison of differential expression between ALS-C9orf72-wild-type vs. controls and bulbar onset ALS-C9orf72-expanded vs. controls. (Fig C) Correlation of posteriors after SVA correction. Correlation of probabilities in the validation set for NSC, SVM and LASSO after SVA correction labelled as ALS (x-axis) or control (y-axis). Whereas the probabilities for all classifiers where highly correlated, LASSO was free of any bias introduced by the SVA label. The top row displays the results for ALS vs. controls and the bottom row for ALS vs. mimics. (DOCX) [file pone.0198874.s001.docx]

## Whole blood transcriptome analysis in amyotrophic lateral sclerosis: a biomarker study

W. van Rheenen et al.

## Supporting information file content:

- Table A: Diagnoses for ALS mimics
- Fig A: Tissue enrichment for differentially expressed genes
- Fig B: Comparison of differentially expressed genes for sub-phenotypes
- Fig C: Correlation of posteriors after SVA correction

| **Diagnosis** | **n** |
| --- | --- |
| Benign fasciculations | 9 |
| Myelopathy | 8 |
| Spinal muscular atrophy | 8 |
| Chronic inflammatory demyelinating polyneuropathy | 6 |
| Polyneuropathy | 5 |
| Hereditary spastic paraplegia | 4 |
| Neuralgic amyotrophy | 4 |
| Functional | 4 |
| Myopathy | 3 |
| Radiculopathy | 3 |
| Mononeuropathy | 3 |
| Myasthenia gravis | 2 |
| Multifocal motor neuropathy | 2 |
| Bulbospinal muscular atrophy (Kennedy’s syndrome) | 2 |
| Plexopathy | 1 |
| Distal SMA | 1 |
| Segmental SMA | 1 |
| Radiation neuropathy | 1 |
| Facial onset sensory and motor neuronopathy | 1 |
| Hyper CK-emia | 1 |
| Primary progressive multiple sclerosis | 1 |
| Corticobasal degeneration | 1 |
| Developmental disorder | 1 |
| Fear for ALS | 1 |
| Unexplained, spontaneous recovery | 2 |
| Total | 75 |

Table A Diagnoses for ALS mimics.

| 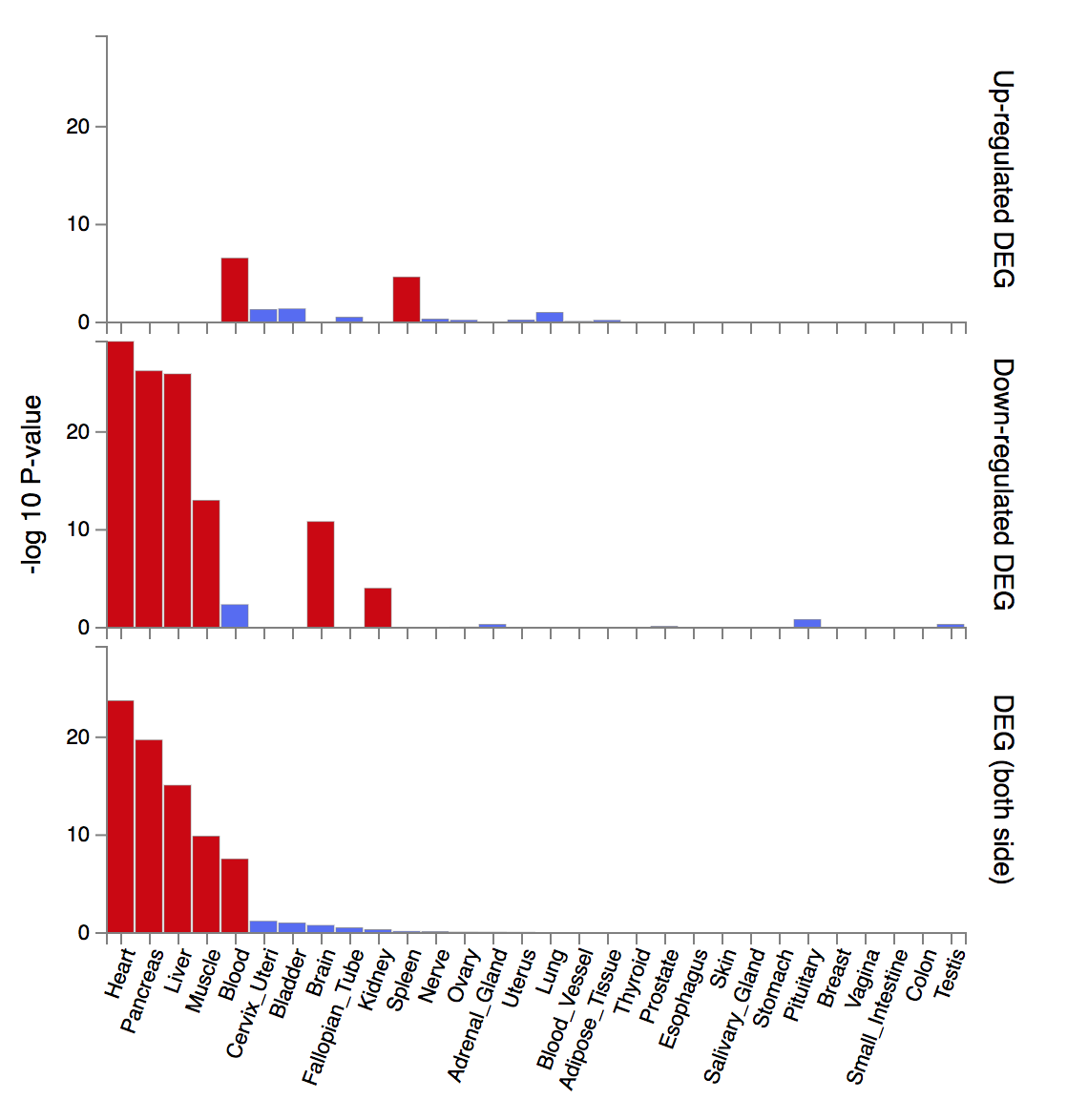 |
| --- |
| **Fig A Tissue enrichment for differentially expressed genes (FUMA).** Top: p-values for differentially upregulated genes per tissue when compared to expression levels in all other tissues. Middle: p-values for differentially downregulated genes per tissue when compared to expression levels in all other tissues. Bottom: p-values for differentially expressed (up- and downregulated) genes per tissue when compared to expression levels in all other tissues. |

|  |
| --- |
|  |
| **Fig B Differential expression for sub-phenotypes.** Top: comparison of differential expression between spinal onset ALS vs. controls and bulbar onset ALS vs. controls. Bottom: comparison of differential expression between ALS-C9orf72-wild-type vs. controls and bulbar onset ALS-C9orf72-expanded vs. controls. |

####

**Fig C Correlation of posteriors after SVA correction.** Correlation of probabilities in the validation set for NSC, SVM and LASSO after SVA correction labelled as ALS (x-axis) or control (y-axis). Whereas the probabilities for all classifiers where highly correlated, LASSO was free of any bias introduced by the SVA label. The top row displays the results for ALS vs. controls and the bottom row for ALS vs. mimics.
